# Do altered energy metabolism or spontaneous locomotion ‘mediate’ decelerated senescence?

**DOI:** 10.1111/acel.12318

**Published:** 2015-02-26

**Authors:** Oge Arum, John Alexander Dawson, Daniel Larry Smith, John J Kopchick, David B Allison, Andrzej Bartke

**Affiliations:** 1 Department of Internal Medicine, Southern Illinois University-School of MedicineSpringfield, IL, 62794, USA; 2 Section on Statistical Genetics, Department of Biostatistics, School of Public Health, University of Alabama at Birmingham1665 University Blvd, RPHB 140J, Birmingham, AL, 35294-0022, USA; 3 Nutrition Obesity Research Center, Department of Nutrition Sciences, University of Alabama at BirminghamBirmingham, AL, 35294-0022, USA; 4 Department of Biomedical Sciences, Edison Biotechnology Institute, Heritage College of Osteopathic Medicine, Ohio UniversityAthens, OH, 45701, USA

**Keywords:** physiology of longevity, gas-exchange (indirect calorimetry) metabolism, spontaneous physical activity, growth hormone hormonal signaling, caloric restriction, mediation analysis

## Abstract

That one or multiple measures of metabolic rate may be robustly associated with, or possibly even causative of, the progression of aging-resultant phenotypes such as lifespan is a long-standing, well-known mechanistic hypothesis. To broach this hypothesis, we assessed metabolic function and spontaneous locomotion in two genetic and one dietary mouse models for retarded aging, and subjected the data to mediation analyses to determine whether any metabolic or locomotor trait could be identified as a mediator of the effect of any of the interventions on senescence. We do not test the hypothesis of causality (which would require some experiments), but instead test whether the correlation structure of certain variables is consistent with one possible pathway model in which a proposed mediating variable has a causal role. Results for metabolic measures, including oxygen consumption and respiratory quotient, failed to support this hypothesis; similar negative results were obtained for three behavioral motion metrics. Therefore, our mediation analyses did not find support that any of these correlates of decelerated senescence was a substantial mediator of the effect of either of these genetic alterations (with or without caloric restriction) on longevity. Further studies are needed to relate the examined phenotypic characteristics to mechanisms of aging and control of longevity.

## Introduction

There are numerous hypotheses of shared (global, public) mechanisms that modulate aging, and most are derived from studies of genetic alterations that affect one outcome of aging – the ultimate survivorship. While some are potentially important for constructing a mechanistic comprehension of the regulatory dynamics of senescence, gerontological research could benefit from identification of traits associated with the multiple slow-aging phenotypes resulting from a variety of anti-aging interventions and life-extending gene mutations; these traits might be useful as reliable surrogates for lifespan outcomes or credible biomarkers suggestive of extended healthspan.

Measurements estimating the general rate of metabolic processes have long-been correlated with ultimate survivorship and have been proffered as sufficient to explain the rate of senescence (Rubner, 1908). These measurements include heart rate, respiration rate, core body temperature, and the rate of mitochondrial processes such as oxygen utilization/consumption, adenosine triphosphate production, or oxidant generation (Holmes *et al*., 2001; Speakman, 2005). These suggestive mechanisms do not apply broadly, so investigations of the correlations of multiple measures of metabolism with longevity induced by genetic alteration and/or dietary intervention would be of significant interest. Identifying metabolic phenotypes that transcend a particular genetic background or mode of delaying and/or decelerating aging also would be important for proposing mechanisms of extended lifespan and healthspan.

Hormonal somatotrophic signaling-deficient mice are the best-documented examples of genetically determined delayed/decelerated senescence in mammals. Both hypopituitary Ames dwarf mice, homozygous for the hypomorphic *df* point mutation allele of the *prophet of Pit1, paired-like homeodomain transcription factor* (*PROP paired-like homeobox 1*) (*Prop1*^*df*^) gene, which are deficient in growth hormone (GH) production, and Laron syndrome-modeling *growth hormone receptor/binding protein* (*Ghr/bp*) gene-disrupted (GHR-KO) mice are shorter and lighter than their littermate controls. Ames dwarf longevity was the original example for genetic regulation of mammalian lifespan (Brown-Borg *et al*., 1996), while GHR-KO mouse longevity (Coschigano *et al*., 2000, 2003) confirmed that the effect on ultimate survival was due to perturbed GH signaling. This effect is not limited to one gender, genetic background, or diet composition (Bartke *et al*., 2013). Consistent with their increased longevity, Ames dwarfs, endocrinologically similar Snell dwarfs, and GHR-KO mice exhibit a delayed presentation of aging-consequent phenotypes – neoplastic disease, memory impairment, immune system and collagen aging, and neuromusculoskeletal frailty – which supports their characterization as slow-aging animals (Bartke *et al*., 2013).

Dietary restriction paradigms such as caloric restriction (CR), which consists of calorie reduction without malnutrition, have long-been shown as capable of retarding various aspects of the syndrome of senescence. Particularly, CR has been documented to increase life expectancy in experimental organisms ranging from the single-celled fungus *Saccharomyces cerevisiae* to murines, carnivores, and likely also primates (McCay *et al*., 1935; McDonald & Ramsey, 2010). With numerous examples of its manifold aging-retarding effects, CR is the preeminent paradigm of interventions that can counter aging-resultant decline, disorders, and diseases. Curiously, Ames dwarf mice exhibit a gender-independent extension of life expectancy when calorically restricted from young-adulthood, while an identical CR regimen in GHR-KO mice produces only a small, female-specific increase in maximal, but not median or mean, survival (Bartke *et al*., 2013).

Both Ames dwarfs and GHR-KO mice have significantly increased oxygen consumption (VO_2_) per unit of body weight (BW) and a significantly reduced respiratory quotient (RQ)/respiratory exchange ratio (RER) (Westbrook *et al*., 2009), as well as reduced body temperature (Hunter *et al*., 1999; Hauck *et al*., 2001; reviewed in Bartke & Westbrook, 2012). Moreover, there is evidence associating extended longevity with increased VO_2_/g and reduced RQ in mice with targeted deletion of the *growth hormone-releasing hormone* (*Ghrh*) gene and, consequently, isolated growth hormone deficiency (Arum, Westbrook, and Bartke, unpublished). Interestingly, we have detected opposing metabolic alterations (*i.e*., a reduction in O_2_ consumption and an increase in RQ) in ‘giant’ transgenic mice overexpressing GH, in which the growth rate and adult BW are increased and longevity reduced (Westbrook *et al*., 2009). Evidence suggests that mitochondria's altered function and the resulting metabolic adaptations are among key mechanisms that control aging and may represent the final output of signaling pathways that determine longevity (Cheng & Ristow, 2013). Reduced RQ is a marker of increased reliance on lipids, as opposed to carbohydrates, as an energy substrate, and increased fat utilization was associated with extended longevity in *Caenorhabditis elegans* (Wang *et al*., 2008). Yet, research in *Drosophila melanogaster* suggests that metabolic rate does not correlate with individual lifespan, nor is it altered by either of two longevity-inducing interventions: reduced insulin/insulin-like growth factor signaling or dietary restriction (Hulbert *et al*., 2004). Whether the mechanisms by which CR retards senescence include alterations (particularly, decreases) in metabolism has been an active research hypothesis for some time (Ramsey *et al*., 2000).

Mediation analysis is a mode of statistical analysis employed to dissect a known relationship (*X*→*Y*) by exploring the underlying mechanism or process by which independent variable *X* influences dependent variable *Y through* another variable [the mediating variable, or mediator (*M*)] (*X*→*M*→*Y*) (Baron & Kenny, 1986). A mediation relationship occurs when *M* plays a quantifiably important role in governing the relationship between *X* and *Y*. Thus, an established mediator variable serves to clarify the nature of the relationship between independent and dependent variables. As it is the objective of multiple lines of modern experimental gerontology to determine or elucidate the means by which a mutation, diet, drug, or lifestyle intervention engenders retarded aging (including ancillary consequences such as longevity), statistical analyses such as mediation might prove very useful for gerontological research.

With these considerations, we conducted a study to test the following null hypothesis: No individual representative measure of gas-exchange metabolism or spontaneous locomotion measured during the second half of the expected lifespan [mediating variable (*M*)] in aged (∼20–32 month-old) mice will be sufficiently correlated with GH signaling deficiency (with CR as an important covariate) [independent variable (*X*)] and ultimate survivorship [dependent variable (*Y*)] such that *M* can be established as a ‘mediator’ of the effect of *X* on *Y*.

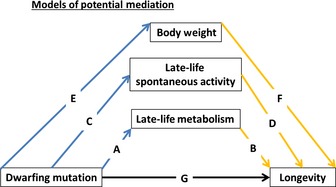



## Results

### Longitudinally measured physiological parameters of study cohorts

Lower growth rates and adult body weights/sizes of mutant mice relative to their littermate controls were consistently observed across stocks and sexes (Fig. S1A–D, *P*-value < 0.001 for each stock- and sex-specific comparison of effect of phenotype). Similarly, moderate CR had weight gain-attenuating effects for stocks and sexes tested (Fig. S1A–D, *P*-value < 0.001 for each stock- and sex-specific comparison of effect of diet). Furthermore, significant lifespan extensions with CR but not the Ames Df mutation (*P* = 0.05 and *P* = 0.22; see Fig. S1E), and in the GHR-KO mutant but not with CR in that stock (*P* = 0.005 and *P* = 0.12; see Fig. S1F), were statistically inferred under Aalen survival models (see Methods).

### Metabolic alteration as a mediator?

**Fig 1:**
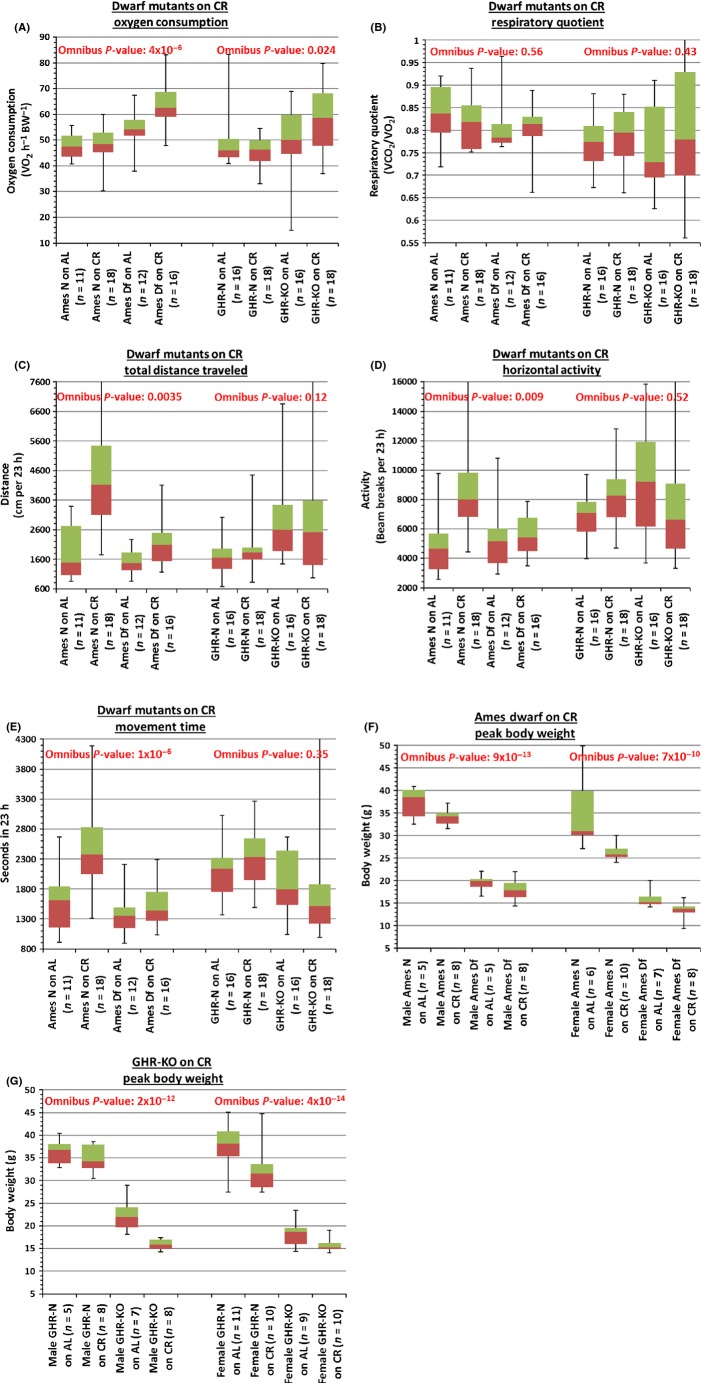
Anatomical and Physiological Characteristics. (A) Gender-independent light-phase survivorship oxygen consumption (VO_2_). (B) Gender-independent light-phase respiratory quotient (RQ). (C) Gender-independent total distance (cm.). (D) Gender-independent horizontal movement count (beam breaks). (E) Gender-independent movement time (s). (F) Maximum body weight (B.W.) for Ames dwarfs. G. Maximum body weight (B.W.) for GHR-KO mice. (Legend: N = littermate control (normal) mice, Df = Ames Dwarfs, KO = GHR-KO mice, AL = *ad libitum* diet, CR = 30% caloric restriction diet).

**Fig 2:**
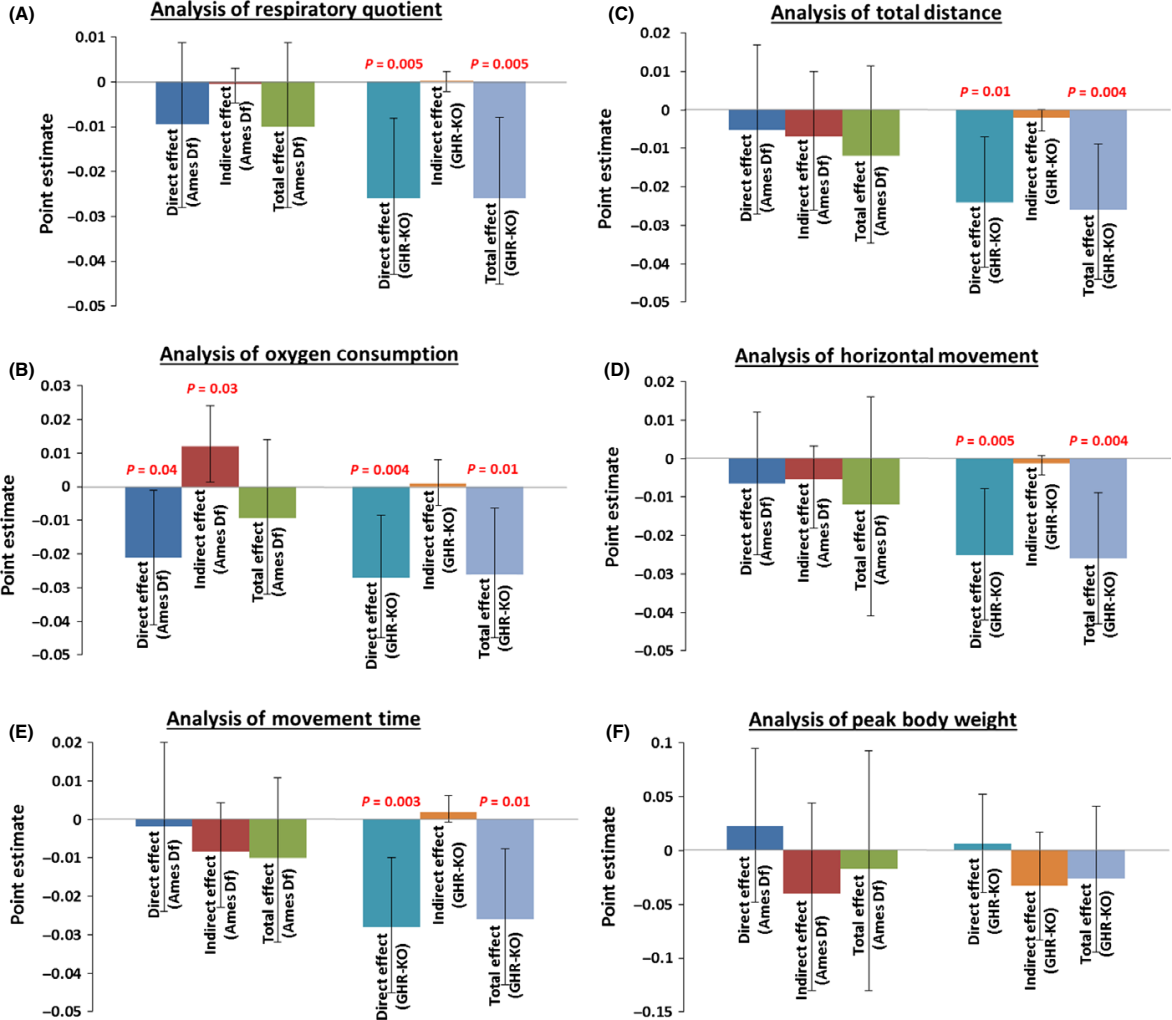
Results of Mediational Analyses. (A) Analysis of oxygen consumption (VO_2_). (B) Analysis of respiratory quotient (RQ). (C) Analysis of total distance (cm). (D) Analysis of horizontal movement count (beam breaks). (E) Analysis of movement time (s). (F) Analysis of maximum body weight (B.W.).

Neither oxygen consumption (VO_2_ per gram of lean body mass) nor respiratory quotient (RQ = VCO_2_/VO_2_), both measured during the light phase of the 12:12 h light:dark cycle to provide an estimate of resting (or, more accurately, less-active) energy expenditure, could be concluded as a mediating variable of the beneficial effects of Ames dwarfism (± CR) or *Ghr/bp* gene disruption (± CR) on ultimate survivorship (Ames Df stock: RQ *P*-value = 0.82; GHR stock: VO_2_ *P*-value = 0.77, RQ *P*-value = 0.97 (Figs1A,B and 2A,B; Table S1). Correspondingly, energy expenditure (oxygen consumption or RQ) during the dark phase (more-active portion) was not a statistically significant mediator (data & analysis not shown), leading to the conclusion that none of these metabolic measures is a mediator of longevity in any of the three models.

### Spontaneous locomotor activity as a mediator?

Late-life spontaneous locomotor activity is often used as a behavioral marker of delayed and/or decelerated senescence (Ingram, 2000; Manini, 2010; Minor *et al*., 2011; Wilkinson *et al*., 2012; Neff *et al*., 2013; Zhang *et al*., 2013). This unforced activity serves as a surrogate for the level of functionality of the multiple health dimensions that would permit it; animals with musculoskeletal pain or disability, respiratory distress or disability, easy fatigability, generalized hyperalgesia, low energy due to poor nutrient intake or assimilation, pain or weakness due to malignant neoplastic disease(s), or any of a number of other domains of the multifactorial syndrome of frailty (Walston *et al*., 2006) would presumably be disinclined to move or cover less area when trying.

Total distance (the total distance the animal traveled), horizontal activity counts (total number of disruptions in horizontal photobeams), and movement time (the length of time the animal spent in either ambulatory or stereotypical activity), all measured over a 24-h period, were not significant mediating variables of increased survivorship [Ames Df stock: total distance *P*-value = 0.42, horizontal activity *P*-value = 0.25, movement time *P*-value = 0.205; GHR-KO stock: total distance *P*-value = 0.085, horizontal activity *P*-value = 0.29, movement time *P*-value = 0.18 (Figs1C–E and 2C–E; Table S1)].

### Body weight as a mediator?

In a study of CR effects on a group of recombinant inbred lines, greater loss of weight (Liao *et al*., 2010) and/or adiposity (Liao *et al*., 2011) was inversely correlated with survivorship. Moreover, it stands to reason that the majority of weight-deficient mutants are shorter-lived rather than long-lived; as maintaining tissue integrity (and thus body weight) at the evolutionarily set level is a gross hallmark of health. This c*aveat emptor* notwithstanding, BW was tested as a mediator variable of the beneficial effects of dwarfism (± CR) on survivorship. Reduced GH hormonal signaling leads to reduced body size by reduced support of postnatal tissue proliferation, along with obesity, as GH is lipolytic; thus, *while body weight is down, obesity is up in these mutants*. Conversely, CR with a postdevelopmental onset reduces weight by reducing tissue proliferation and increasing the catabolism of adipose depots for energy. Despite their differing means, both interventions reduce BW and increase survivorship (among many other outcomes they independently engender). Therefore, we sought to determine whether maximum BW [the heaviest balance-determined weight of an individual mouse during the study period (Fig.1F,G)] could serve as a mediating variable (one for which there is greater pre-existing substantiation than for metabolic or behavioral outcomes) within our study.

Peak BW was not a mediating variable in the relationships of either the Ames *df* mutation (± CR) or the *Ghr/bp* gene disruption (± CR) to longevity [Ames Df *P*-value: 0.35; GHR-KO *P*-value: 0.2 (Figs1F,G and 2E; Table S1)]. Similar (nonsignificant) results were obtained when BW at age of metabolic testing was assessed as a potential mediator.

## Discussion

In mice, GH resistance and GH deficiency are associated with remarkable extensions of healthy lifespan. Caloric restriction can cause a further increase in longevity and healthspan in some of these long-lived mutants. When examined in early adulthood or middle-age, long-lived mice exhibit a number of changes in energy metabolism. However, the significance of these physiological and metabolic alterations in mediating the health promotion and mortality reduction remains unclear. While longevity studies remain a staple of model organism aging research, identification of reliable biomarkers of aging as intermediate surrogates of reduced mortality could significantly reduce the time and costs associated with aging research. In the present study, none of a series of metabolic characteristics measured, even when measured in advanced age, was significantly and consistently related to longevity across 16 genotype/diet/gender groups, and thus none of these measures could be identified as a mediator of the effects of reduced GH signaling ± CR on aging.

Insufficient power might have contributed to some or all of the negative results of our mediation analyses. While it is not straightforward to simulate indirect effects (since both the *X*→*M* and *M*→*Y* relationships need to be specified and, hence, there are a lot of specifications to be made), as a sensitivity analysis we performed some post-*hoc* power calculations, given the same within-stock sample sizes and using the same lifespan means and spreads. Assuming that increased longevity is fully mediated through an intermediate process such as RQ, with these sample sizes we would be 80% powered to find the indirect effect significant if the effect sizes for each of the aforementioned relationships were about 0.9; given that full mediation of genetic mutations is unlikely in biological pathways, this represents an observable effect size floor; hence, our belief that insufficient power may have contributed to our negative results. Additionally, we cannot consider more than one mediator at a time in this framework. However, if there were some sort of consistent relationship between light-phase (resting) VO_2_ and RQ, we probably would have seen some marginal effects. That we did not countermands arguments that (potentially substantial) interactive or combinatorial effects of individual variables preclude the establishment of any one such variable as a mediator.

It is worth noting that GHR-KO females are reported to have innately decreased spontaneous locomotion when measured at 17 months (Longo *et al*., 2010), potentially as a measure to reduce their energetic output, thus offsetting the increased energetic demands of their higher surface area-to-body volume ratios (Longo *et al*., 2010). If spontaneous locomotion of the employed long-lived mutants, particularly our Ames dwarfs, is lower than that of their normal littermates before old age, the present documentation of indistinguishable levels of activity between these mice and the normal controls at an older age [Figs1C–E and2C–E; Table S1, (at ∼ 22 months of age: O. Arum & A. Bartke, unpublished)] could be interpreted as evidence for protection from the aging-associated decline in spontaneous activity.

As the metabolic and activity measures are collected from singly housed animals (to accurately measure animal-specific metabolic outcomes) while longevity outcomes are assessed under co-housed conditions, the possibility exists that changes in cage-mate-affected bioenergetic or social conditions could influence either the metabolic rate or activity patterns.

The potentially confounding effects of associating a phenotype measured under a standard set of conditions with a phenotype assessed under more-eliciting conditions (such as how single-housing conditions would be expected to influence metabolic outcomes moreso in leaner mice than in fatter ones) on our search for metabolic or spontaneous activity mediators are also recognized.

Maximum BW failing to be determined as a mediator of life expectancy is likely due to the increased adiposity that results from decreased GH action (Berryman *et al*., 2004), which muddles the expected results of diminished GH signaling (^1^'smaller' animals that ^2^live longer) by increasing the body weight of animals genetically destined for longevity. The intuitive importance of reduced adiposity for increased life expectancy is similarly countermanded by the documentation that calorically restricted mice that maintain a higher degree of adiposity outlive their leaner CR counterparts (Rikke *et al*., 2010; Liao *et al*., 2011). Therefore, in some circumstances, heightened adiposity is not detrimental to, and might actually contribute to, retained health and survivorship.

In conclusion, our results fail to support mechanistic hypotheses asserting any of the examined parameters of metabolism or locomotion as substantial mediators of aging or longevity. These characteristics, while not being *bona fide* mediators in the strict, statistical sense, may represent biological markers of processes that *are* causally related to aging (such as ‘mitochondrial efficiency' or ‘fat burning’).

## Experimental procedures

See Supplemental Experimental Procedures for detailed information on animal husbandry, indirect calorimetry measurement, and spontaneous activity assessment.

### Statistical methodology

When a measurement is associated with an outcome and it can be considered to come before the outcome (either logically or chronologically), it is often of interest to ask how much of the measurement's effect on the outcome can be explained via its effects on an intermediate variable. Specifically, given an association between a predictor *X* and an outcome *Y* and an association between *Y* and another predictor *M*, a mediational analysis quantifies the extent to which the association between *X* and *Y* is *mediated* through *M* (which is, in turn, called a *mediator*) by partitioning the *total effect* of *X* on *Y* into a *direct effect* (the effect of *X* on *Y* given *M*) and an *indirect effect* (the effect of *X* through *M* on *Y*).

To get an intuitive feel for this concept, consider these examples: In areas where malaria is common, mosquito bites are associated with increased mortality. However, the direct effect of said bites is nil; the increased mortality is almost fully mediated through the introduction of *Plasmodium* parasites. In contrast, cancer patients who undergo chemotherapy have decreased mortality and incur hair loss, but there is nil improvement in survival through the hair loss *per se*; hence, hair loss is not a mediator of improved survival (*i.e*., nil indirect effect).

Our results are stratified based on stock (Ames Df or GHR-KO) and for each stock the following are recorded: mutation status (heterozygous or homozygous mutant; binary); diet (AL or CR; binary); sex (binary); and maximum observed body weight. Additionally, the following are recorded based on hourly measurements taken over a 24-h span, covering one light and one dark cycle: oxygen consumption (averaged over the span); respiratory quotient (averaged over the span, after first replacing biologically implausible RQ values with lower or upper 1% percentiles as appropriate); total distance travelled (summed over the span); horizontal activity (summed over the span); and movement time (summed over the span). Light- or dark-cycle-specific mediators were obtained by averaging or summing only over the span of 07:00–18:00 or its complement, respectively.

As a quick note, there were 130 animals in the experiments but five were removed from the analyses because their health was failing at the time of the measurements and they died within a week of their measurements; hence the total numbers of animals observed across the plots and in the text is 125.

In these mediational analyses, our outcomes are survival times, which may be censored. As such, many of the more common mediational approaches are not applicable, as they do not take the presence of censoring into account. Therefore, our potential mediators were assessed in accordance with the approach of Lange & Hansen (2011); the reader may find it useful to peruse a contemporary commentary by VanderWeele (2011). As such, we are assuming an Aalen additive hazards model when regressing survival outcomes on predictors (both marginally as in Fig. S1E,F and as part of the mediational analyses), while using linear regression for intermediate regressions in the mediational analysis. Mutation status, sex and diet were included as covariates during the calculations of the direct and indirect effects. Confidence intervals and corresponding *P*-values were likewise determined by bootstrap, as directed by Lange & Hansen (2011). We do not consider nominally significant direct or indirect effects to be significant if there is not also a significant total effect, but all results are reported for completeness. All *P*-values presented are nominal (unadjusted for multiple comparisons), which we take into account when discussing the results. All mediational analyses were implemented in R (The R Development Core Team, 2012). Mediational analyses were directly coded, rather than utilizing a pre-existing R package, although we made use of elements from the survival package.

For line plots, measures of central tendency are arithmetic means, and all depictions of variation (error bars) represent standard deviations (SD). For box-and-whisker plots, horizontal lines in boxes denote 75th, 50th (median), and 25th percentiles (from top to bottom), and upper and lower whiskers denote maxima and minima, respectively. For bar plots, bars represent point estimates from the mediation analysis, and depictions of variation (error bars) represent confidence intervals (C.I.).

## Supporting Information

**Data S1.** Individual-level Values from Indirect Calorimetry & Spontaneous Activity Experiments.

**Data S2.** Hour-level Values from Indirect Calorimetry & Spontaneous Activity Experiments.

**Data S3.** Supplemental Experimental Procedures. **Fig. S1.** Anatomical and physiological characteristics. **Table S1.** Results of mediational analyses.
