# Supplementary material for: Do altered energy metabolism or spontaneous locomotion ‘mediate’ decelerated senescence?
Source: Aging Cell. 2015 Feb 26;14(3):483–90. doi: 10.1111/acel.12318 (PMC4406677; doi:10.1111/acel.12318)
Supplement: Supplementary file 3 [file acel0014-0483-sd3.docx]

**SUPPLEMENTAL DATA**

**SUPPLEMENTAL FIGURE 1.**

**Anatomical and Physiological Characteristics.** A. Body weight trajectory for Ames dwarf males. B. Body weight trajectory for Ames dwarf females. C. Body weight trajectory for GHR-KO males. D. Body weight trajectory for GHR-KO females. E. Gender-independent survivorship plot for Ames dwarfs. F. Gender-independent survivorship plot for GHR-KO mice. . (Legend: N = littermate control (normal) mice, Df = Ames Dwarfs, KO = GHR-KO mice, AL = *ad* *libitum* diet, CR = 30% caloric restriction diet)

A.


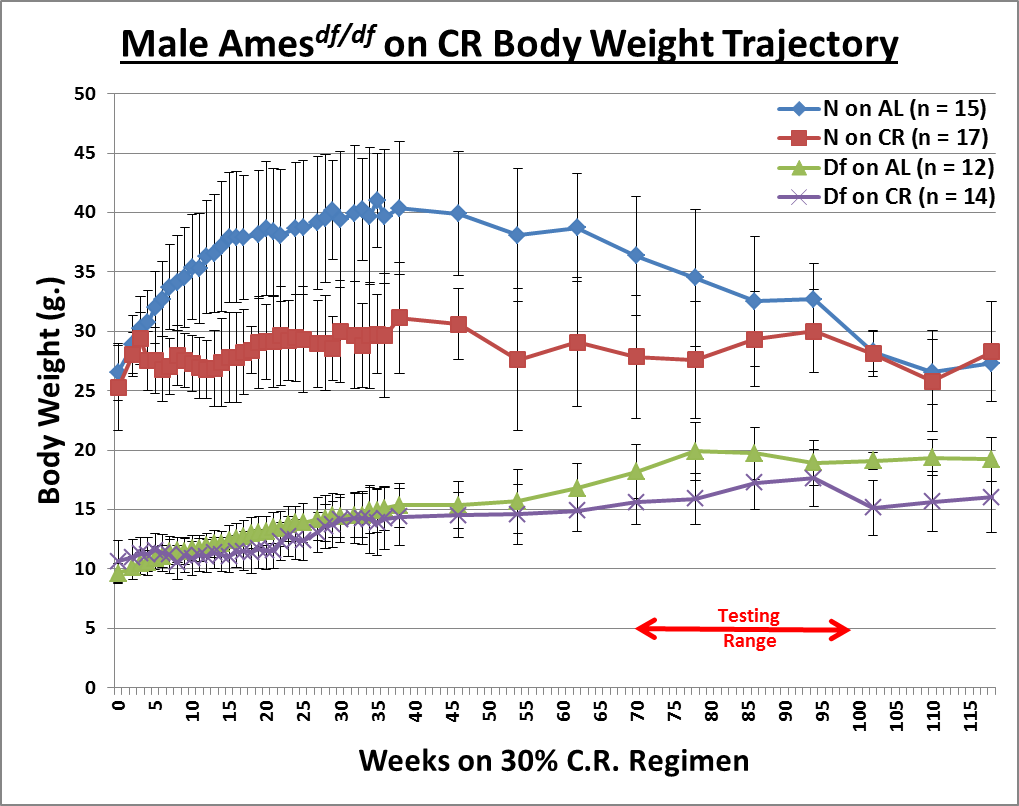


B.


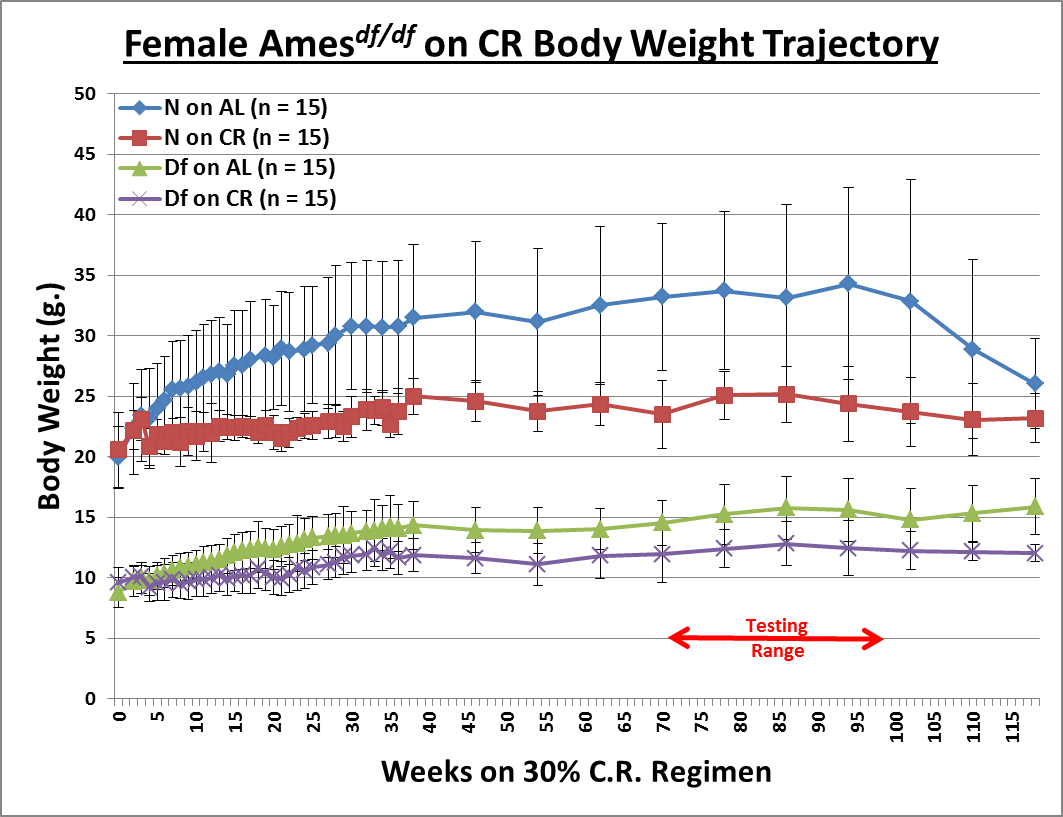


C.


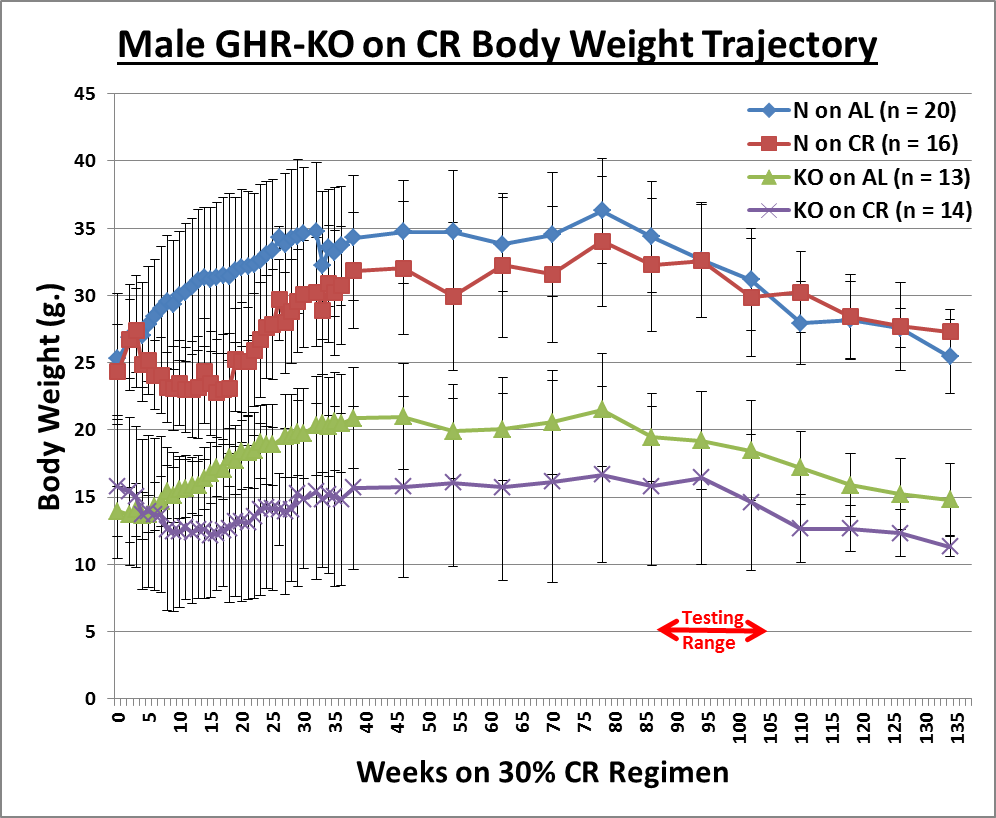


D.


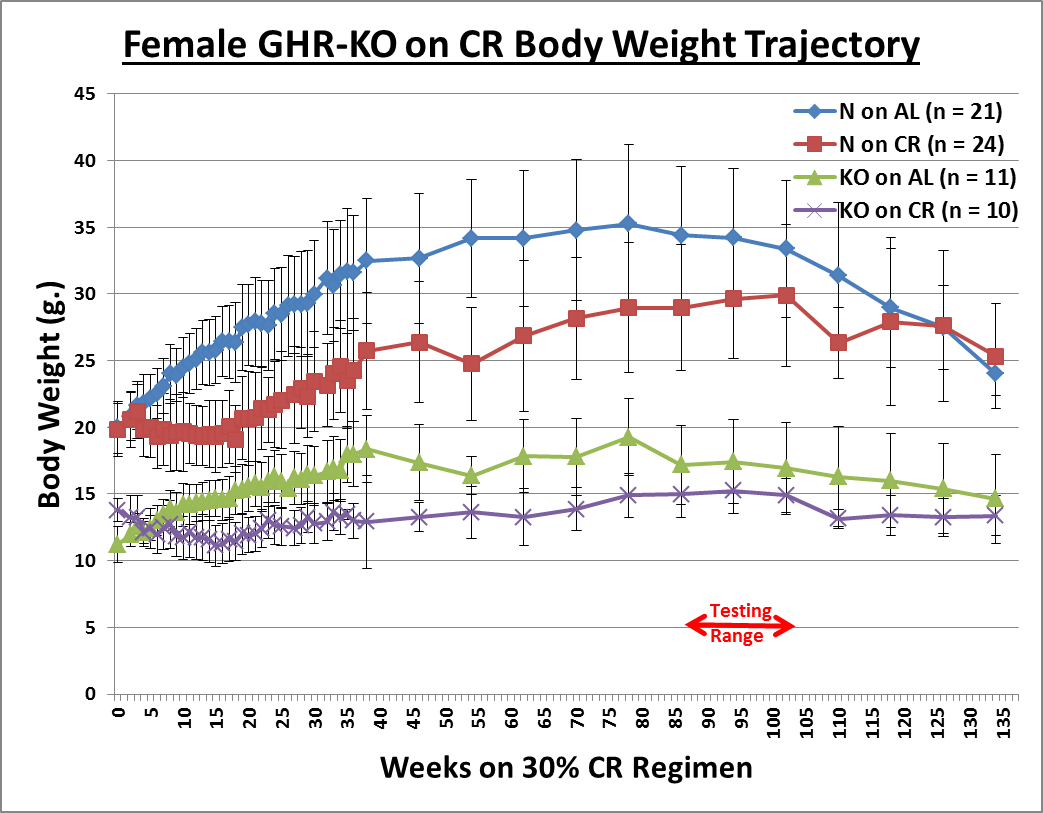


E.


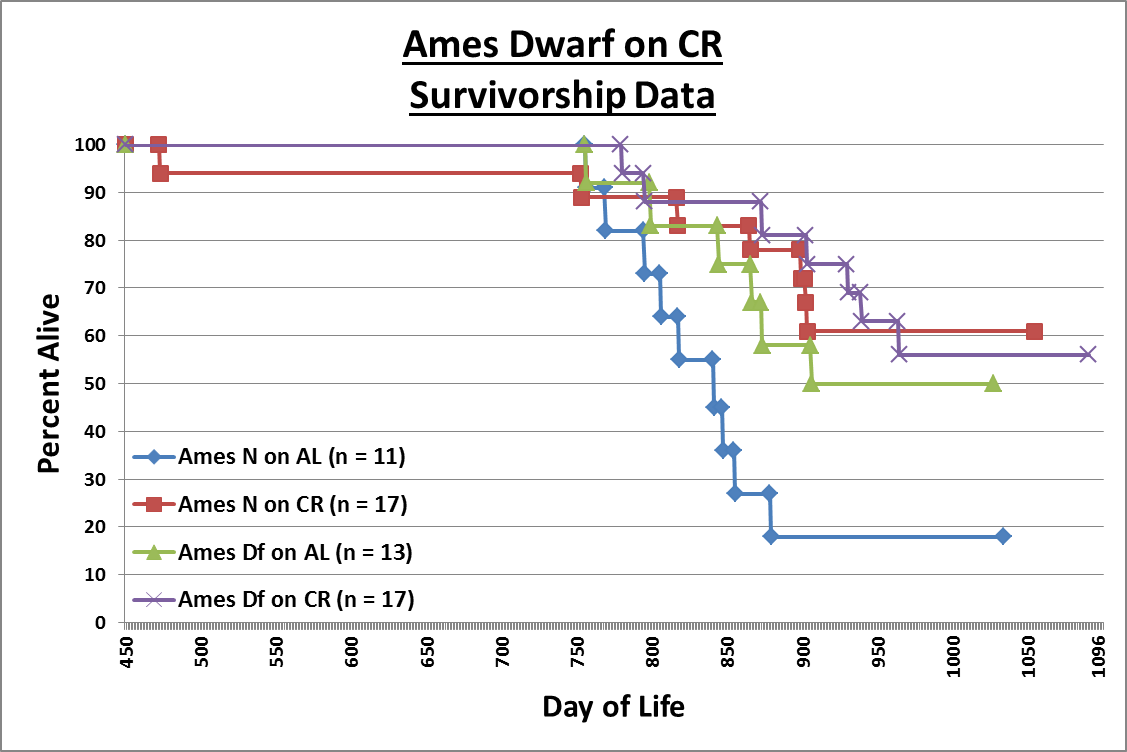


F.


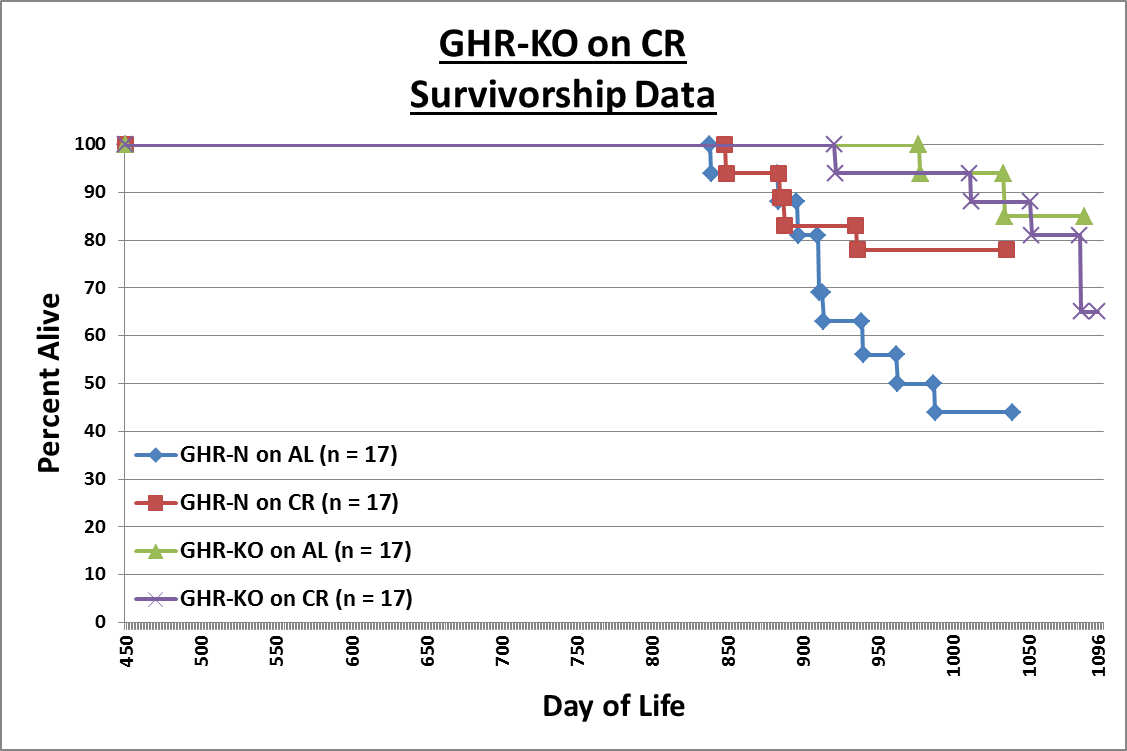


**SUPPLEMENTAL TABLE 1. Results of Mediational Analyses**

| **Ames Df Stock** | Point Estimate | 95% Confidence Interval | *p*-value |
| --- | --- | --- | --- |
| *RQ* |  |  |  |
| Direct effect | -0.0094 | (-0.028, 0.0088) | 0.31 |
| Indirect effect | -0.00041 | (-0.0047, 0.0031) | 0.82 |
| Total effect | -0.0099 | (-0.028, 0.0087) | 0.30 |
|  |  |  |  |
| *VO_2_* |  |  |  |
| Direct effect | -0.021 | (-0.041, -0.0010) | 0.04 |
| Indirect effect | 0.012 | (0.0013, 0.024) | 0.03 |
| Total effect | -0.0094 | (-0.032, 0.014) | 0.425 |
|  |  |  |  |
| *Total Distance* |  |  |  |
| Direct effect | -0.0051 | (-0.027, 0.017) | 0.66 |
| Indirect effect | -0.0069 | (-0.026, 0.0099) | 0.42 |
| Total effect | -0.012 | (-0.041, 0.016) | 0.405 |
|  |  |  |  |
| *Horizontal Mov’t* |  |  |  |
| Direct effect | -0.0065 | (-0.025, 0.012) | 0.49 |
| Indirect effect | -0.0053 | (-0.018, 0.0033) | 0.25 |
| Total effect | -0.012 | (-0.034, 0.0089) | 0.27 |
|  |  |  |  |
| *Mov’t Time* |  |  |  |
| Direct effect | -0.0018 | (-0.024, 0.020) | 0.875 |
| Indirect effect | -0.0083 | (-0.023, 0.0044) | 0.205 |
| Total effect | -0.010 | (-0.036, 0.015) | 0.445 |
|  |  |  |  |
| *Peak Body Weight* |  |  |  |
| Direct effect | 0.023 | (-0.048, 0.095) | 0.52 |
| Indirect effect | -0.040 | (-0.13, 0.044) | 0.35 |
| Total effect | -0.017 | (-0.13, 0.093) | 0.765 |
|  |  |  |  |
| **GHR KO Stock** | Point Estimate | 95% Confidence Interval | *p*-value |
| *RQ* |  |  |  |
| Direct effect | -0.026 | (-0.043, -0.0081) | 0.005 |
| Indirect effect | 0.000050 | (-0.0021, 0.0023) | 0.97 |
| Total effect | -0.026 | (-0.043, -0.0079) | 0.005 |
|  |  |  |  |
| *VO_2_* |  |  |  |
| Direct effect | -0.027 | (-0.045, -0.0085) | 0.004 |
| Indirect effect | 0.00092 | (-0.0056, 0.0081) | 0.77 |
| Total effect | -0.026 | (-0.045, -0.0064) | 0.01 |
|  |  |  |  |
| *Total Distance* |  |  |  |
| Direct effect | -0.024 | (-0.041, -0.0069) | 0.01 |
| Indirect effect | -0.0021 | (-0.0056, 0.00019) | 0.085 |
| Total effect | -0.026 | (-0.044, -0.0088) | 0.004 |
|  |  |  |  |
| *Horizontal Mov’t* |  |  |  |
| Direct effect | -0.025 | (-0.042, -0.0078) | 0.005 |
| Indirect effect | -0.0012 | (-0.0043, 0.00078) | 0.29 |
| Total effect | -0.026 | (-0.043, -0.0088) | 0.004 |
|  |  |  |  |
| *Mov’t Time* |  |  |  |
| Direct effect | -0.028 | (-0.045, -0.010) | 0.003 |
| Indirect effect | 0.0020 | (-0.00064, 0.0062) | 0.18 |
| Total effect | -0.026 | (-0.043, -0.0076) | 0.01 |
|  |  |  |  |
| *Peak Body Weight* |  |  |  |
| Direct effect | 0.0065 | (-0.039, 0.052) | 0.78 |
| Indirect effect | -0.033 | (-0.083, 0.017) | 0.20 |
| Total effect | -0.026 | (-0.094, 0.041) | 0.445 |

Direct and indirect mediational effects and their sum (total effect). The direct, indirect, and total effects are unitless and reflect additive changes to the hazard (force of mortality). Negative values represent effects that improve survival, and all effects are on the same scale and hence comparable.

**SUPPLEMENTAL EXPERIMENTAL PROCEDURES**

**Animal Husbandry**

All animals were bred in closed colonies, housed under standard conditions (12-hr. light/ 12- hr. dark light-cycling and 20-23^o^C temperature), and fed Lab Diet Formula 5001 (23% protein, 4.5% fat, and 6% fiber) (Nestle-Purina, St. Louis, MO). Body weighing, general health assessment, and exclusions for suspect health were conducted as previously described (Arum *et* *al.*, 2013a; Arum *et* *al.*, 2013b). Animals of similar ages were housed 3-5/cage during the testing period. Animal protocols were approved by the Animal Care and Use Committee of Southern Illinois University.

Ames dwarf mice and their heterozygous littermate controls have a unique genetic background, with respect to extant inbred strains of mice; this stock exhibits approximately 25% polymorphism at examined loci (Pawlikowska, L. & Bartke, A., unpublished). GHR-KO mice and their heterozygous littermate controls were derived from 129/Ola founders outbred to Balb/c, provided by Dr. J.J. Kopchick (Ohio University, Athens, OH), and subsequently crossed to a stock derived from C57Bl/6J and C3HJ strains. Although without the methodological benefits of “reproducible genetic heterogeneity” (Miller *et al.*, 1999), these stocks possess considerably more genetic variation (Panici *et al.*, 2009), which correlates with broad-based health and life expectancy, than inbred strains.

Somatotrophic signaling-deficient mice have such a marked difference in snout-to-anus length, being approximately 80% of the length of their littermates, that these animals are grouped by their “dwarf” phenotype. They are not genotyped, as prior genotyping-corroborated experience has proven that it is redundant (Gage *et al.*, 1996). [Importantly, this method of classification obviates the potential confound of using weight as the distinguishing characteristic, as that can be complicated in certain instances because the Ames dwarfs and the GHR-KO mutants (both of which lack lipolytic GH signaling) have increased subcutaneous adiposity (Berryman *et al.*, 2004; Berryman *et al.*, 2010).]

*Caloric Restriction*

The amount of food allotted each cage of mice designated for caloric restriction was determined based on (weekly calculated) *ad libitum* food consumption for entire cages of gender-, genotype-, and birthdate-matched controls, and these values were averaged over the number of cages within each such group. Thusly, we calculated how much food was to be placed in each CR cage’s food-hopper. (The mice were fed daily in the late afternoon.) As mice were not individually housed, we could not calculate how much any particular animal consumed on either diet. As a protection against dissimilar food consumption in CR cages, part of the food was broken into pieces small enough to pass through the hopper grate (but not into crumbs). Observation confirmed that this permitted every restricted mouse to feed *ad libitum* during the initial surge of food consumption. Considering the valid concerns related to differential restriction resulting from a dominant cage-mate consuming relatively more food, we were very attentive to individual mouse weight loss and health (*e.g.* fight wounds indicative of physical conflicts with a cage-mate) through-out our studies. It is also worth noting that our chosen level of restriction (30%) is moderate compared to the 40% level that causes considerable concerns (Liao *et al.*, 2010; Mattson, 2010), and this moderate 30% level does not lead to exhausting food supply after the initial gorge (thus, even subordinate mice would have ample, albeit possibly delayed, access to food).

**Indirect Calorimetry**

The Fusion Metabolic System (AccuScan Instruments Inc., Columbus, OH) comprised a metabolic analyzer, O_2_ analyzer, CO_2_ analyzer, multi-channel flow controller, Windows XP-based Fusion Software, and airtight cages. The Metabolic System measured and stored O_2_ and CO_2_ content and airflow and generated and stored VO_2_, VCO_2_, and RQ data, for each subject in one to eight enclosed environments (chambers). The O_2_ analyzer used a zirconia sensor and had a built-in sample pump and flow controller. The CO_2_ analyzer had a dual-beam sensor and required no calibration. The flow controller had independent pumps for each channel (chamber), as well as a mass flow meter. Flow ranges were set to approximately 0.5 L/min.

Each animal was placed in an airtight chamber with *ad* *libitum* access to food and water. Ambient air was pumped through the cage. The amount of air entering the cage was precisely measured by a mass flow meter. Air entering and leaving each chamber was monitored for O_2_ and CO_2_ content by zirconia and infrared sensors, respectively. Both sensors are more accurate and maintenance-free than electrochemical cells. All experimental data [variables: animal weight (g.), reference flow rate (mL/min.), reference O_2_%, reference CO_2_%, cage flow rate (mL/min.), cage O_2_%, cage CO_2_%, %O_2_ change, %CO_2_ change, VO_2_ (mL/kg./min.), VCO_2_ (mL/kg./min.), respiratory quotient (VCO_2_/VO_2_)] were continuously monitored, displayed, and stored for future analysis; heat production and energy expenditure were calculated thusly:

Heat production (kcal./hr.): [(4.33 x VO_2_) + (0.67 x VCO_2_)] + [Wt.(kg.)] x (60 min./hr.)

Energy expenditure (kJ/min.): VO_2_ x (364 + 113 x RQ)/22.4

An acclimation period of approximately 24 hours preceded the AL-fed day testing. Data were normalized per unit of lean body weight (Ramsey *et* *al*., 2000; Butler & Kozak, 2010), as determined by fat depot sub-dissection of adult mice matched for gender, genotype, and chronological age. [Given the consistency of group-relative differences in body composition observed in prior analyses on adult GHR-KO mice (Berryman *et al.*, 2004; Berryman *et al.*, 2010; Arum & Bartke, unpublished), no direct measures of body composition were acquired for the mice on which other experiments presented in this study were conducted.]

**Spontaneous Activity**

Various measures of activity (total distance, horizontal activity count, ambulatory activity count, rest time, rest episode count, movement time, movement episode count, stereotypy time, stereotypy episode count, stereotypy activity count, vertical episode count, vertical activity count, vertical activity time, locomotor clockwise revolutions, and locomotor counter-clockwise revolutions) were measured coincidently with the indirect calorimetry within the Fusion Metabolic System (AccuScan Instruments Inc., Columbus, OH) by virtue of 16 interlaced photobeams along each of the vertical and horizontal planes.

The hardware detected beams broken by the animal, and the software determined the location of the rodent within the cage. Each monitor consisted of a node attached to the cage frame that had a small unobtrusive profile. Each node captured 100 frames/second from its respective monitor for each of the two sensor pairs (spatial planes). Sensor pairs had 16 evenly spaced infrared light-beams/axis that traversed each chamber.
